# The Seropositivity of *Toxocara* spp. Antibodies in Pregnant Women Attented at the University Hospital in Southern Brazil and the Factors Associated with Infection

**DOI:** 10.1371/journal.pone.0131058

**Published:** 2015-07-06

**Authors:** Paula Costa Santos, Lis Maurente Lehmann, Carolina Lorenzi, Carolina Hirsch, Paula Lima Telmo, Gabriela Torres Mattos, Priscila Silva Cadore, Gabriel Baracy Klafke, Maria Elisabeth Aires Berne, Carla Vitola Gonçalves, Carlos James Scaini

**Affiliations:** 1 Laboratory of Parasitology, Faculty of Medicine—FAMED, Area Interdisciplinary Biomedical Sciences (AICB) Federal University of Rio Grande–FURG Rio Grande, Rio Grande do Sul, Brazil; 2 Obstetric Center, University Hospital of Rio Grande–Rio Grande, Rio Grande do Sul, Brazil; 3 Laboratory of Parasitology, Institute of Biology, Department of Microbiology and Parasitology, Federal University of Pelotas, Pelotas, Rio Grande do Sul, Brazil; Royal Tropical Institute, NETHERLANDS

## Abstract

**Background:**

Human toxocariasis is a parasitic zoonosis with a worldwide distribution but is underdiagnosed with an underestimated impact on human health. The ingestion of embryonated eggs of *Toxocara* spp. present on the hands or in contaminated food or water is the main mode of infection. The only record of *Toxocara* congenital infection in humans occurred in a premature infant. Helminth infections during pregnancy may be associated with reproductive disorders. Studies investigating the occurrence of toxocariasis in pregnancy are scarce, as is research on the possible implications of these parasites in reproductive health. The aim of this study was to determine the seroprevalence of antibodies to *Toxocara* spp. in pregnant women and to identify risk factors associated with its infection.

**Methodology/Principal Findings:**

The cross-sectional study of the seropositivity of specific antibodies for *Toxocara* spp. was performed on 280 pregnant women. Serum samples were examined with enzyme-linked immunoassay. Epidemiological data were obtained through a questionnaire containing information about obstetric history, general life style choices, and the social and economic status of the women. The prevalence of *Toxocara* spp. IgG in pregnant women was 6.4%. Some of the risk factors associated with the infection were owning dogs (p = 0.003), living in the city centre (p = 0.028), living at the city beach (p = 0.003), and having a family income at or below minimum wage (p < 0.001). There was no association between reproductive disorders and *Toxocara* seropositivity.

**Conclusions/Significance:**

The seroprevalence of 6.4% for *Toxocara* spp. in pregnant women shows that there was exposure to the parasite. The study demonstrates the need for attention for the completion of clinical diagnosis parameters, as well as the expansion of highly specific serological studies in different regions to understand the impact of toxocariasis in pregnancy.

## Introduction

Human toxocariasis is a parasitic zoonosis with a worldwide distribution but is underdiagnosed with an underestimated impact on human health [1,2]. However, this disease has been considered the most prevalent helminthiasis in endemic areas in America [3,4]. In addition, the Centers for Disease Control and Prevention (CDC) considers this parasitosis among the five parasitic diseases that require public health actions [5]. Serologic studies in children have shown prevalence rates of greater than 50% [6–7] and in adults the prevalence rate is 8.7% [8] to 44.9% [9].

In Brazil the only study to date in pregnant women, the seropositivity for *Toxocara* spp. was 7.4% [10] though in China was recorded seropositivity 9.19% [11]. The ingestion of embryonated eggs of *Toxocara canis* present on the hands or in contaminated food or water is the main mode of infection [12]. However, cases have been reported involving the consumption of meat and/or raw or undercooked viscera of chicken, ducks and cattle infected with *T*. *canis* larvae [13–15]. The only record of *Toxocara* congenital infection in humans occurred in a premature infant with retinopathy with blood eosinophilia whose mother was seropositive for *Toxocara* spp [16]. This information shows the need for the preparation of health care professionals to diagnose this parasitosis because the diagnosis of this disease is based on the association of symptoms with laboratory and epidemiological data [17–18]. Early diagnosis is considered critical for administering treatment the different clinical forms of human toxocariasis [5].

Helminth infections in pregnancy may be associated with maternal anemia [19], susceptibility to inflammatory diseases and the possibility of effects on fetal immune response [20]. Studies investigating the occurrence of toxocariasis in pregnancy are scarce, as is research on the possible implications of these parasites in reproductive health. Furthermore, there is little specific information on the risk of infection to the mother and the foetus during pregnancy [21]. The aim of this study was to investigate the seropositivity of *Toxocara* spp. specific IgG in pregnant women attended at the University Hospital-Rio Grande (FURG) and the epidemiological factors associated with *Toxocara* spp. infections.

## Methods

### Population study

The cross-sectional study of the prevalence of specific antibodies (IgG) for *Toxocara* spp. was performed on 280 pregnant women from May 2011 to April 2012 attended at the Obstetric Center, University Hospital of Rio Grande—RS, Brazil. The sample size was calculated using Epi Info 3.5.2, software (CDC, Atlanta, Georgia, USA) expected prevalence at 7.4 [10], to evaluate a 95% degree of confidence, a tolerated error of 5% and 10% losses.

#### Participation of the pregnant women

The women were invited to participate in the study. The patient participation was contingent on signing the Informed Consent Form (ICF) (S1 File) which included authorising the analysis of a blood sample collected during routine prenatal care and access to medical records and answering an epidemiological questionnaire. For pregnant women less than 18 years old, a legal guardian was asked to sign the ICF. The information contained in the questionnaires, medical records and results of the survey will be confidential and only available to researchers involved in it, to protect the identity of the research subjects. This study was approved by the Ethics Committee on Research in Health–FURG (CEPAS n° 33/2011 23116.001226/2011-97).

### Epidemiological data

A structured questionnaire was administered in the maternity ward, University Hospital, after parturition by two trained researchers. The questionnaire contained questions concerning obstetric history (history of abortion, prematurity, number of pregnancies and parturitions), general life style choices (age, onicophagy, local of residence, living with pets, type of food eaten), and the social and economic (living arrangements, income level, educational level) status of the women. The questionnaires were double-entered using EpiData 3.1 software (Odense, Denmark).

#### Research on medical records

In the medical records data of the pregnant women, the eosinophilia levels, results of laboratory diagnosis of other infectious and parasitic diseases (Acquired Immune Deficiency Syndrome, hepatitis, toxoplasmosis) and child birth weights were surveyed.

### Serum samples of pregnant women

A blood sample from each patient was collected in The Clinical Analysis Laboratory of the University Hospital. After obtaining the serum sample, it was aliquoted into an tube and stored at 20°C for later analysis.

#### Excretory-secretory antigens (TES) production

*Toxocara canis* eggs were collected from female adult parasites after treating young dogs with pyrantel pamoate (15 mg/kg). The eggs were then incubated in a 2% formalin solution at 28°C and oxygenated for 30 days [22]. Hereafter, the larvae derived from embryonated eggs were incubated 37 °C, 5–8% CO_2_ in RPMI-1640 medium with antibiotics and antifungals [23] to obtain excretory-secretory antigens (TES) of the infective *T*. *canis* larvae [24]. The determination of the protein concentration was performed using the Bicinchoninic Acid Method (BCA) [25].

#### Somatic antigen of *Ascaris suum* (SoAs)

This antigen was produced from adult female *A*.*suum* acquired from a slaughterhouse in the city of Pelotas, Rio Grande do Sul, Brazil, following the methodology described by Souza *et al*. (2011) [26]. The determination of the protein concentration of this antigen was performed using BCA [25].

#### Serology

Serum samples were pre-adsorbed with the SoAs antigen [26]. To conduct research on the IgG anti-*Toxocara* spp. antibodies indirect ELISA were performed using the TES (2 μg/mL) antigen in carbonate/bicarbonate buffer. The free binding sites were blocked with 5% casein in PBS/Tween-20 0.05% (PBS-T), sera were diluted 1:50 in PBS/Tween and peroxidase conjugates and anti-human IgG (Fc specific) (1:7000) (Sigma Aldrich, San Diego, CA, USA). The chromogen used was orthophenylenediamine (OPD) [27]. Each sample of serum was examined in duplicate using a 450 nm wavelength. Samples of seropositive children to *Toxocara* spp. was used for positive controls. The *cut off* point (0.205) was established from the mean absorbance of the thirty three negative control sera (with eosinophilia below 3% and without contact with dogs) plus three standard deviations.

### Statistical analysis

To determine the association between the seropositivity for *Toxocara* spp. and the sociodemographic, obstetric, dietary habits and contact with animal data, the categorical variables were analysed using the chi-square test. The prevalence ratio was calculated for each variable and was considered a significant difference *p*<0.05. Multivariate analysis was performed using logistic regression, followed by construction of a multivariable hierarchical linear model that incorporated variables with *p*≤0.20 in the crude analysis. The first level consisted of demographic and socioeconomic variables (family income and domicile), and the second level consisting of the risk factors for *Toxocara* spp. (domiciled dog, cat domiciled and vegetable consumption). All analyses were performed with Epi Info 3.5.1 software (CDC, Atlanta, Georgia, USA).

## Results

Of the 280 serum samples analysed by ELISA-TES, the anti-*Toxocara* spp. immunoglobulin (IgG) seropositivity was 6.43% (18) in these pregnant women.

In the bivariate analysis of the social and demographic characteristics of the study population, family income and place of residence were observed to be risk factors for infection with *Toxocara* spp. (Table 1).

**Table 1:** The seropositivity (IgG) for *Toxocara* spp. according to sociodemographic data of the pregnant women attended at the University Hospital in Rio Grande-RS, Brazil from May 2011 to April 2012 (n = 280).

Variable	Samples		Positivity		Prevalence Ratio	Confidence interval CT 95%	*p* value
	N	(%)	N	(%)			
**Age**							0.431
13–19	58	(20.7)	05	(8.6)	1		
20–24	82	(29.3)	06	(7.3)	1.21	0.46–3.10	
25–29	73	(26)	04	(5.5)	0.81	0.27–2.38	
30–34	41	(14.6)	02	(4.9)	0.73	0.17–3.05	
35 or more	26	(9.4)	01	(3.8)	0.57	0.08–4.14	
**Family Income (Minimum Wage)**							**<0.001**
3 or more	78	(27.8)	01	(1.3)	1		
Up to 2	146	(52.1)	02	(1.4)	0.11	0.02–0.48	
≤ 1	56	(20.0)	15	(26.8)	20.0	5.9–66.70	
**Domicile**							**0.014**
Periphery	180	(64.3)	08	(4.4)	1		
Downtown	36	(12.8)	03	(8.3)	1.35	0.41–4.45	
BalnearyCassino	18	(6.4)	04	(22.2)	4.15	1.52–11.3	
Rural	29	(10.4)	02	(6.9)	1.12	0.27–4.64	
Another municipality	17	(6.1)	01	(5.9)	0.97	0.12–7.77	

(Chi-square, *p* ≤0,05)

Regarding the bivariate analysis of the risks of infection, we found that 88.9% and 94.4% of seropositive pregnant women had owned dogs (*p*<0.001) and consumed vegetables (*p* = 0.03), respectively (Table 2).

**Table 2:** The seropositivity (IgG) for *Toxocara* spp. according to the epidemiological factors in the pregnant women attended at the University Hospital of Rio Grande—RS, from May 2011 to April 2012(n = 280).

Variable	Sample		Positivity		Prevalence Ratio	Confidence interval CI 95%	*p value*
	N	(%)	N	(%)			
**Domiciled dog**							**0.001**
No	134	(47.9)	02	(1.5)	1		
Yes	146	(52.1)	16	(11)	7.45	1.75–31.81	
**Contact with dog faeces**							0.822
No	231	(82.5)	15	(6.5)	1		
Yes	49	(17.5)	3	(6.1)	0.94	0.28–3.13	
**cat ownership**							0.092
No	224	(80)	17	(7.6)	1		
Yes	56	(20)	1	(1.8)	0.24	0.03–1.73	
**Onychophagia**							0.395
No	196	(70)	11	(5.6)	1		
Yes	84	(30)	7	(8.3)	1.48	0.59–3.69	
**Meat consumption raw and / or undercooked**							0.615
No	182	(65)	13	(7.1)	1		
Yes	98	(35)	5	(5.1)	0.71	0.26–1.94	
**Consumption of processed food (sausage)**							0.422
No	30	(10.7)	3	(10)	1		
Yes	250	(89.3)	15	(6)	0.60	0.18–1.95	
**Raw Vegetable consumption**							**0.03**
No	75	(26.8)	1	(1.3)	1		
Yes	205	(73.2)	17	(8.1)	6.38	0.86–47.16	
**Contact with sand**							0.755
No	193	(68.9)	13	(6.7)	1		
Yes	87	(31.1)	5	(5.7)	0.85	0.31–3.31	
**Attends squares and/or parks**							0.366
No	97	(34.6)	8	(8.2)	1		
Yes	183	(65.4)	10	(5.5)	0.66	0.27–1.62	

(Chi-square, *p* ≤0,05)

Table 3 demonstrated there was no significant difference between the seropositive and seronegative pregnant women in relation to reproductive disorders and the presence of blood eosinophilia.

**Table 3:** The seropositivity (IgG) for *Toxocara* spp. according to the obstetric history and the blood eosinophilia status in the pregnant women attended at the University Hospital of Rio Grande—RS, from May 2011 to April 2012 (n = 280).

Variable	Sample			Positivity		Prevalence Ratio	Confidence interval CI 95%	*p value*
		N	(%)	N	(%)			
**Abortion**								0.771
No		220	(78.6)	15	(6.8)	1		
Yes		60	(21.4)	3	(5.0)	0.73	0.21–2.45	
**Reporting Difficulty conceiving**								0.443
No		248	(88.6)	15	(6.8)	1		
Yes		32	(11.4)	3	(9.4)	1.55	0.47–5.06	
**History of premature parturition**								0.302
No		240	(85.7)	14	(5.8)	1		
Yes		40	(14.3)	4	(10)	1.71	0.59–4.94	
**History of low birth weights**								0.302
No		240	(85.7)	14	(5.8)	1		
Yes		40	(14.3)	4	(10)	1.71	0.59–4.94	
**Eosinophilia**								0.675
No		262	(93.6)	17	(6.5)	1		
Yes		18	(6.4)	1	(5.6)	0.91	0.12–6.47	

(Chi-square, *p* ≤0,05)

Of the women interviewed, only 28.9% (81) had taken some preventive measure against parasites during pregnancy (*p* = 0.602). Furthermore, only 0.7% (2) claimed to have knowledge of human toxocariasis, and these patients were negative for *Toxocara* spp.

In the multivariate analysis, the following variables were identified as independent risk factors for infection *Toxocara* spp.: ownership of a domiciled dog (PR = 9,68; CI = 2.15–43.53; *p* = 0.003), family income at or below minimum wage (PR = 25.41; CI = 6.71–96.16; *p* ≤ 0.001), and residing in downtown (PR = 6.64; CI = 1.23–35.77; *p* = 0.028) or in Balneary, Rio Grande (PR = 10.8; CI = 1.22–92.99; *p* = 0,003).

## Discussion

In toxocariasis, there are limitations to determining a clinical diagnosis because of the lack of specific symptoms and the lack of a gold-standard laboratory diagnostic method. These factors, along with the lack of epidemiological surveys, hinder achieving definitive diagnosis parameters, transmission control or a cure for this neglected parasitosis. In this study, the serum samples were examined using ELISA-TES, which is considered the standard method in clinical laboratories and experimental trials. The seroprevalence of 6.43% for *Toxocara* spp. in pregnant women shows that there was exposure to the parasite. This information demonstrates the need for studies in this risk group.

The prevalence found in this study was lower than that observed by Taylor *et al*. (1996) [21], which detected rate of 35.3% in pregnant women in the United States, and In a study conducted in hospital in China was 9.19% (91/990).[11], and higher than the 4.5% found by Gasanova *et al*. (2003) [28] in a population of pregnant women in Russia. However, our prevalence rate was similar to the prevalence of 7.4% that was recorded by Pereira (2007) in a study of pregnant women in Brasilia-Federal District [10].

The low prevalence in pregnant women might be explained by hemodilution that occurs during pregnancy due to the increase in plasma volume generated by the high demand of placental tissue. This hemodilution can hinder the detection of immunoglobulins in the serum. Moreover, it is possible that transplacental IgG migration occurs. The transport of IgG starts from the sixteenth week of pregnancy and is continuously increasing after the twenty-second week until the foetus presents IgG levels similar to those of an adult [29,30].

It is also important to note that the prevalence rates may vary depending on the population studied and the laboratory method employed. In the studies conducted in Brazil or in other developing countries with a tropical climate, pre-adsorbing the serum with antigens from other helminths is necessary to avoid cross-reactions, especially with *Ascaris* spp. [31,7].

Furthermore, the close contact between humans and dogs is an important risk factor for infection by *Toxocara* spp. [6,32]. Of the positive pregnant women, 88.9% had owned dogs, which indicates that dog ownership is an important risk factor in pregnant women for infection with *T*. *canis* (*p* = 0.001).

The dog population in urban areas is a risk factor for the infection of humans because of the potential for soil contamination with parasite eggs [33]. In the present study, we observed association between seropositivity against *Toxocara* spp. with pregnant women residing in the city centre of Rio Grande (*p* = 0.028) and in the balneary Cassino (*p* = 0.003). In a study of stool samples from dogs collected in balneary Cassino, contamination was observed in 86.1% of samples with larvae and/or helminth eggs, 9.3% being eggs of *Toxocara* spp., which demonstrates that the population is exposed to a risk of infection [34].

Embryonated eggs of *Toxocara* spp. are resistant to environmental conditions and are among the major contaminants of soil [12]. In a previous study, Manini *et al*. (2012) showed that there was a significant relationship between soil contamination and positive serology for *Toxocara* spp. in children and that environmental contamination by *Toxocara* spp. is an important risk factor for infection [33]. The results indicate the need for prevention measures, such as control of the dog population and instituting responsible ownership guidelines to reduce the environmental pollution [35].

The analysis of socioeconomic characteristics revealed a significant association between income at or below minimum wage and the risk for *Toxocara* infection. The seropositivity for *Toxocara* spp. was associated with a low socioeconomic status of the study population. A serological survey in Brasilia found a seropositivity of 21.8% in children treated in the health care system and 3% in those that use a private service. The populations with lower incomes are also devoid of a health infrastructure, making this population more exposed to infection [36].

Of the pregnant women with seropositivity for *Toxocara* spp., 55.6% reported the occurrence of some reproductive disorder (abortion, difficulty getting pregnant, premature birth), but there were no significant differences between this and the uninfected group. In relation to *Toxocara* seropositivity and abortions, there are controversial conclusions. Similarly, to our results, Pereira (2007) noted that in pregnant women with a IgG seropositivity against *Toxocara* spp.,21.7% had at least one abortion, but this was not significant as a risk factor [10]. On the other hand, Taylor et al. (1996) found a statistically significant relationship between seropositivity against *Toxocara* IgG and the occurrence of previous abortions. They found that 35% of seropositive pregnant women had a history of previous abortions compared with 8.6% of serologically negative (p = 0,044). The authors discuss the possibility of a migration of larvae by host tissues and organs may cause tissue damage that is associated with the occurrence of abortion [21].

Regarding the level of blood eosinophils in the present study, no association with seropositivity was found, which was also observed in a study conducted in children [37]. However, Roldán *et al* (2008) showed a significant association with eosinophilia in the population of school children in North Lima, Peru. The authors discussed that human toxocariasis can cause eosinophilia accompanied or not of symptoms [38].

Despite the registration of a congenital infection in human toxocariasis [16], the Centers for Disease Control and Prevention does not consider this parasite to be transmitted during pregnancy [5]. However, there is evidence of vertical transmission by *T*. *canis* and *T*. *cati* in definitive hosts (dogs and cats) [39], and *T*. *canis* larvae in parathenic hosts demonstrate the capacity and tropism to migrate during pregnancy [40–41] or lactation to offspring, reinforcing the importance of conducting studies in this area in humans [42–43].

## Conclusions

The exposure of pregnant women to *Toxocara* spp. (6.43%) demonstrates the need for attention for the completion of clinical diagnosis parameters, as well as the expansion of highly specific serological studies in different regions to understand the impact of toxocariasis in pregnancy.

## Supporting Information

S1 File Informed Consent Form. (PDF)
