# Supplementary material for: The Seropositivity of Toxocara spp. Antibodies in Pregnant Women Attented at the University Hospital in Southern Brazil and the Factors Associated with Infection
Source: PLoS One. 2015 Jul 6;10(7):e0131058. doi: 10.1371/journal.pone.0131058 (PMC4492739; doi:10.1371/journal.pone.0131058)
Supplement: S1 File — (PDF) [file pone.0131058.s001.pdf]

FURG

FEDERAL UNIVERSITY OF RIO GRANDE

FACULTY OF MEDICINE

GRADUATE PROGRAM IN HEALTH SCIENCES

HEALTH SCIENCES MASTER DEGREE COURSE

Informed Consent Form

Name:____Number:________

Prevalence of antibodies against *Toxocara canis* in pregnant women at the University Hospital of Rio Grande

This work will be performed by students of the Master in Health Sciences, Paula dos Santos Costa, under the supervision of Dr. Carlos and Dr. James Scaini. Carla Vitola Gonçalves with the aim of determine the possible presence of antibodies against disease toxocariasis pregnant women. This study was approved by the Ethics Committee on Research in Health–FURG (CEPAS n° 33/2011 23116.001226/2011-97).

Participation consists in permission of serological analysis of the blood sample collected at admission, to respond to a questionnaire on the obstetric history and socioeconomic status, and access to data from the medical charts.

The pregnant woman's blood sample will be obtained in the prenatal routine, with no need for special collection for this study, not posing a risk to pregnant women. Women who agree to participate will not be identified with the samples will be numbered in the laboratory, the test result shall be reported to the pregnant woman or her legal guardian, by telephone or mail, using the contact provided for completing the questionnaire and also the treatment where this is required, which is already standardized by the National Health System will be available.All data obtained in the survey, and the results will only be available to participants and researchers involved in it. The identity of the participants will not be exposed in any publication.

Received explanations about the study reported in this consent form. I was able to clarify my doubts, and all my questions were answered clearly. I declare to agree to participate voluntarily in this study, knowing that I have the right to stop participating at any time, without any prejudice or loss of any right.

I understand that does not exist remuneration for researchers or participants of this research.

I,____, RG nº___________, declare that I have been informed and agreed to the participation as a volunteer in the above research project.

Rio Grande, _____ de ____________ de 201___.

____

signature

Special cases of consent: Patient under 18 - with the assistance of a parent or guardian; Patient and / or guardian illiterate - this document should be read aloud to the patient and the guardian in the presence of two witnesses, who also will sign the document;

I,____,RG nº ___________, legal representative by __________________________________, RG nº _________________, declare that I have been informed and agree with your participation as a volunteer in the above research project.

____

signature

Research Coordinator: Prof. Dr. Carlos James Scaini

Laboratory of Parasitology, Faculty of Medicine - FAMED, Area Interdisciplinary Biomedical Sciences (AICB) Federal University of Rio Grande – FURG Rio Grande, Rio Grande do Sul, Brazil.

phone number: (53) 3233.8871 / 32338887
